# Single Step Nanoplasmonic Immunoassay for the Measurement of Protein Biomarkers

**DOI:** 10.3390/bios3010077

**Published:** 2013-02-06

**Authors:** Shradha Prabhulkar, Adam de la Zerda, Amit Paranjape, Richard M. Awdeh

**Affiliations:** 1 Department of Ophthalmology, University of Miami-Bascom Palmer Eye Institute, Miami, FL 33136, USA; E-Mails: sprabhulkar@med.miami.edu (S.P.); asparanjape@gmail.com (A.P.); 2 Department of Structural Biology, Stanford University, Palo Alto, CA 94305, USA; E-Mail: adlz@stanford.edu

**Keywords:** gold nanorods, optical coherence tomography, protein biomarkers, immunoassay, surface plasmon resonance, glucose transporter-1

## Abstract

A nanoplasmonic biosensor for highly-sensitive, single-step detection of protein biomarkers is presented. The principle is based on the utilization of the optical scattering properties of gold nanorods (GNRs) conjugated to bio-recognition molecules. The nanoplasmonic properties of the GNRs were utilized to detect proteins using near-infrared light interferometry. We show that the antibody-conjugated GNRs can specifically bind to our model analyte, Glucose Transporter-1 (Glut-1). The signal intensity of back-scattered light from the GNRs bound after incubation, correlated well to the Glut-1 concentration as per the calibration curve. The detection range using this nanoplasmonic immunoassay ranges from 10 ng/mL to 1 ug/mL for Glut-1. The minimal detectable concentration based on the lowest discernable concentration from zero is 10 ng/mL. This nanoplasmonic immunoassay can act as a simple, selective, sensitive strategy for effective disease diagnosis. It offers advantages such as wide detection range, increased speed of analysis (due to fewer incubation/washing steps), and no label development as compared to traditional immunoassay techniques. Our future goal is to incorporate this detection strategy onto a microfluidic platform to be used as a point-of-care diagnostic tool.

## 1. Introduction

Immunoassays have been used in research and clinical settings over the past five decades to quantify biomolecules of various sizes, chemical and physical properties [1]. The most commonly used immunoassays based on the principle of antigen-antibody selective binding and labeling are radio-immunoassays, enzyme-linked immunoassays, luminescent immunoassays, and fluorescent immunoassays [2,3,4]. We suggest an alternative immunoassay using antibody-conjugated gold nanorods (Ab-GNRs) as the label and optical coherence tomography (OCT) as the detection mechanism. 

Noble metal nanostructures are of great interest for optical imaging due to their remarkable capacity to absorb and scatter light at visible and near-infrared (NIR) regions, due to the conversion of photon energy to surface plasmon resonance [5,6]. These optical properties depend on nanoparticles size, shape, and dielectric environment, enabling their application as novel imaging and sensing probes [7]. They have excellent biocompatibility, are non-toxic, and are not susceptible to photobleaching [8,9,10]. The collective, in-phase oscillations of conductive electrons in metallic nanoparticles are known as surface plasmon or particle plasmon resonance. This collective electron oscillation causes considerable local-field enhancements at the resonance frequency of the particle plasmon [11]. The principle of using OCT for gold nanorod (GNR) detection relies on the local surface plasmon resonance effect caused by the incident OCT light [12,13]. GNRs are preferred over any other shape of particles as their plasmon resonant wavelength can be precisely tuned to the central wavelength of the OCT beam [14]. GNRs also have very high quality factors and surface area, which translates to large local-field enhancements [15]. Spherical gold nanoparticles have also been used to develop colorimetric and aggregation [16,17,18,19] based biosensors for the detection of proteins in biological samples. 

OCT is a non-contact imaging technology that produces high-resolution cross-sectional images of the internal microstructure of living tissue by detecting backscattered and backreflected light. OCT relies on low-coherence interferometry to measure time delay and intensity of back scattered light [20]. Briefly, a beam splitter halves the light from a low-coherence source, sending one-half towards the tissue and the other-half towards a reference mirror. The backscattered and backreflected light from the sample and the reference mirror then travels back to the beam splitter. In this way the same light source interacts with both the sample and reference mirror and the two resulting beams interfere with each other before producing a waveform on the photodetector [21]. The hardware components of the OCT system, which consists of a broadband light source, Michelson interferometer, and photodetector, are relatively simple and lost-cost. 

We have used the Glut-1 (Glucose transporter-1) protein as our model analyte towards immunosensor development. The Glut protein family, which consists of 14 hexose transporters, is responsible for the facilitated transport of glucose between the extra- and intracellular environment. Glut-1, which is responsible for maintaining basal glucose levels, is ubiquitously expressed in most cells. Elevated expression of Glut-1 has been observed in cancer cells due to their altered metabolic rates, high rates of glucose consumption, and glycolysis. Increase in glucose consumption helps supply energy for tumor cell proliferation and adaptation to the hypoxic tumoral microenvironment. Glut-1 is overexpressed in many tumors, including breast, pancreatic, hepatic, esophageal, brain, renal, lung, cutaneous, endometrial, ovarian, and cervical cancers [22,23,24]. Glut-1 overexpression is also linked to increased proliferative activity, energy requirements, aggressive behavior, and poor prognosis [25,26]. 

## 2. Experimental Section

### 2.1. Materials

Cetyl trimethylammonium bromide (CTAB), chloroauric acid (HAuCl_4_), sodium borohydride (NaBH_4_), silver nitrate (AgNO_3_), ascorbic acid, benzyldimethylammoniumchloride hydrate (BDAC), 11-mercaptodecanoic acid, polyacrylic acid (PAA, M.W. ~15,000), and 3,3',5,5' tetramethylbenzidine (TMB), MES buffer (pH = 6) were purchased from Sigma-Aldrich (Saint-Louis, MO, USA). SH-PEG-COOH bidirectional polymer was obtained from Nanocs (New York, NY, USA). 1-Ethyl-3-[3-dimethylaminopropyl] carbodiimide (EDC), N-hydroxysulfosuccinimide (NHSS) and rabbit anti-Glut-1 polyclonal antibodies were purchased from Thermo Fisher Scientific Inc. (Rockford, IL, USA). Human Glut-1 protein, and Human vascular endothelial growth factor (VEGF) were purchased from Alpha Diagnostics International Inc. (San Antonio, TX, USA). Horseradish peroxidase labeled anti-rabbit secondary antibodies were purchased from Abcam (Cambridge, MA, USA). Ninety-six well high-binding microtiter plates were purchased from Greiner Bio-One (Monroe, NC, USA). 

### 2.2. Preparation of Gold Nanoparticles/Nanoseeds

Two milliliters of 0.20 M CTAB solution was sonicated with 2 mL of 0.5 mM HAuCl_4_ solution for a period of 10 min at a temperature of 40 °C until the solution is clear. Once the solution was cooled down to room temperature, 240 µL of 0.01 M ice-cold NaBH_4_ was added to it and stirred for 2 min. The final brownish-yellow seed solution was stored at 25 °C until further use.

### 2.3. Preparation and Characterization of GNRs

In order to synthesize GNRs displaying longitudinal wavelengths of 840 nm, 12 µL of gold nanoseed solution was added to growth solution containing 5 mL of 0.2 M CTAB, 50–200 µL of 0.0040 M AgNO_3_, 5 mL of 0.0010 M HAuCl_4 _and 70 µL of 0.0788 M of ascorbic acid. The volume of the AgNO_3_ solution can be adjusted to obtain GNRs of a certain aspect ratio. 120 µL of seed solution was added to the growth solution maintained at a temperature of 28 °C and allowed to equilibrate for 3 h. The GNRs synthesized were collected using 10,000 rpm for 15 min and washed twice using DI water to remove excess growth reagents and spherical/smaller nanoparticles [14,27,28,29,30]. The yield and aspect ratios of the GNRs was determined using transmission electron microscopy (TEM), acquired with a Philips CM-10 TEM (Philips, Eindhoven, Netherlands) operating at 100 kV, 0.3 nm resolution (point-to-point), 18× to 450,000× magnification range. TEM grids were prepared by placing 1 µL of the GNRs solution in a 400-mesh Formvar-coated copper grid and evaporating the solution at room temperature. 100–150 GNRs were analyzed per grid using ImageJ to calculate the mean aspect ratio of the GNRs. The extinction spectra of the GNRs samples through each stage of experiments were measured using a Spectronic 200 UV-NIR spectrophotometer (Thermo Fisher Scientific, Inc.) in the wavelength range between 400 and 1,000 nm. 

### 2.4. Surface Modification of GNRs

Commonly, two distinct strategies are utilized for the attachment of biomolecules to the GNRs surface namely electrostatic adsorption and covalent attachment. Electrostatic adsorption can be achieved by matching the isoelectric point of the antibody and the pH of the CTAB coated GNR solution [31]. However, these physisorbed biomolecules are not strongly bound to the GNR surface and a significant amount get detached during centrifugation used for particle collection and washing [32]. In order to covalently attach biomolecules to the surface of the GNRs, functionally active groups need to be developed on the surface of the GNRs. To develop carboxylic groups on the surface of the GNRs, PAA monomers were polymerized and electrostatically bound onto to the surface of the GNRs using the following procedure. Briefly, 1 mL of GNRs solution was diluted to 10 mL with Milli-Q water. To this solution, 100 mL of 2 mg/mL of PAA dissolved in a 6 mM NaCl solution was added drop-wise, and the resulting solution was stirred vigorously for 6 h [33]. At the end of each activation process the mixture was centrifuged at 15,000 rpm for 30 min to remove unbound molecules and the GNR pellet was then re-suspended in 2 mL of MES buffer. 

### 2.5. Anti-Glut-1 Immobilization on the Surface of Modified GNRs

Using the aforementioned surface modification strategy, carboxylic groups were generated on the surface of GNRs. Thereafter, 500 µL of 0.1 M EDC and 500 µL of 0.1 M NHSS were added to 5 mL of modified GNRs re-suspended in pH = 6 MES buffer. The GNRs were collected by centrifugation after 60 min of activation. The GNR pellet was then introduced into a 1 mL solution 50 µg/mL anti Glut-1 monoclonal antibodies and allowed to interact overnight at 4 °C. Excess reaction solution and unbound antibodies were separated by two rounds of centrifugation, at 10,000 rpm, for 20 min. The pellet containing antibody bound GNRs was suspended in 1× PBS buffer containing 0.1% bovine serum albumin (BSA) and 0.1% normal goat serum (NGS) to block non-specific sites. Zeta potential measurements were conducted on the GNRs dispersed in DI water after each stage of the Ab-GNR assembly. 

### 2.6. Optimization of Antibody Immobilization Protocol

In order to determine the optimal procedure for anti-Glut-1 antibody and GNR conjugation, the supernatant solution generated during the centrifugal separation of Ab-GNR and unbound Glut-1 antibodies, was evaluated using NanoDrop optical spectrophotometer at 280 nm. The evaluation of the supernatant solution was done to measure the amount of unbound antibody leftover after the antibody GNR linking procedure. 

### 2.7. Immunoassay Plate Preparation

40 µL of Glut-1 protein solutions were incubated in each well for 1 h at room temperature, followed by an overnight incubation at 4 °C. High binding 96 well microtiter plates, with hydrophilic surface treatment to enhance the binding of proteins were used as the substrate for the immunoassay. Glut-1 binds to the hydrophilic surface of the well-plates via passive adsorption, similar to the mechanism used in traditional ELISA. The excess protein was then discarded and 1% BSA was added to the wells and incubated for 30 min at room temperature to block any non-specific binding sites. The plates were then washed twice using 1× PBS for 5 min each time to remove any residual BSA solution. The Glut-1 coated plates were then incubated with 100 μL of 30 nM Ab-GNR solution for a period of 45 min at room temperature. The plates were then rinsed thrice with 1× PBS to remove any unbound and loosely bound Ab-GNRs. 100 µL of pH = 14, 1× PBS solution was then introduced into each well and the plate was sonicated at 30 °C for 60 min. 

Ab-GNR constructs generated were analyzed using a secondary anti-rabbit antibody tagged to horseradish peroxidase. TMB was used as the calorimetric substrate and analysis was performed using the Fluostar Optima plate reader with an emission filter at 450 nm. 

### 2.8. OCT Imaging of Immunoassay Plate

The OCT imaging of the sonicated immunoassay plate containing GNRs dispersed in PBS solution was conducted using the Bioptigen Spectral Domain Ophthalmic Imaging System (SDOIS). The light source of the OCT systems is a superluminescent diode, centered at a wavelength of 840 nm, with a bandwidth of 100 nm. Each well was imaged using a rastor scanning protocol covering an area of 1.3 × 1.3 mm. Every imaging session consisted of 500 A scans and 1 B-scans. The distance between the top edge of the well plate and the OCT probe is kept constant at 3.6 cm, while imaging each well.

### 2.9. OCT Image Processing

Two hundred frames were captured for a single B-scan. These frames were then averaged in order to obtain a speckle and background noise free OCT image. The signal intensity was calculated by choosing a region of interest (ROI) and averaging the intensity of every pixel within it. The ROI consisted of 25 × 25 pixel area and is located at a distance of 5 cm from the top edge of the averaged image. The background signal intensity was calculated by choosing a region of interest (ROI) at a distance of 0.5 mm from the top edge of the image. 

### 2.10. Specificity Studies

Following the experimental protocol utilized for Glut-1 detection, we incubated 500 ng/mL of Glut-1, VEGF and BSA respectively in 10 microwells. In order to study the interference that could be caused by non-specific proteins in the test sample we incubated test wells with combinations of Glut-1 + VEGF, Glut-1 +BSA and Glut-1 + VEGF +BSA such that the combined molarity of the samples was always 500 ng/mL. The wells were then twice rinsed for 5 min using 1× PBS washes and then each of the wells was incubated with 100 uL of 30 nM anti-Glut-1 GNR construct solution for a period of 45 min, at room temperature. The wells were then thrice rinsed with 1× PBS to remove any unbound and loosely bound GNR tagged antibodies. 100 µL of pH = 14, 1× PBS solution was then introduced into each well and the plate was sonicated at 30 °C for 60 min. OCT imaging of the sonicated solution present in the microwells was conducted using the Bioptigen Spectral Domain Ophthalmic Imaging System (SDOIS).

## 3. Results and Discussion

### 3.1. Gold Nanorod Fabrication and Characterization

**Figure 1:**
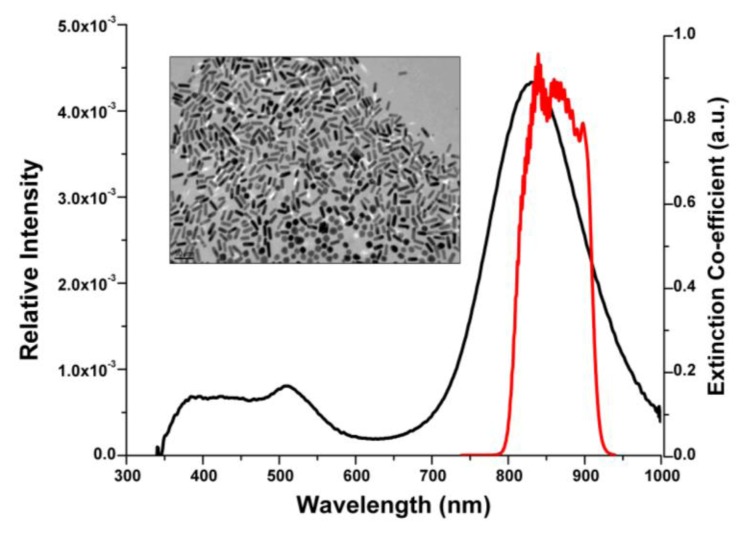
Extinction spectrum of gold nanorods (GNRs) (black line) and relative intensity of (optical coherence tomography) (OCT) imaging light (red line). Inset: transmission electron microscopy (TEM) image of bare GNRs corresponding to longitudinal peak of 840 nm.

GNRs were selected as the indicator label for the immunoassay due to their tunable aspect ratios and high quality factor [6,15]. The tunability of optical resonances is of crucial importance, where nanostructures have to be configured to a selected wavelength such as the central wavelength of the illumination source. The dimensions of the GNRs are closely related to their light scattering and absorbing capabilities. The working principle of OCT is related to the capture and analysis of light backscattered from sample, thus we are interested in preferentially scattering nanoparticles [12]. The extinction spectra of the GNRs is characterized by two peaks, the larger/dominant peak corresponding to the longitudinal surface plasmon resonance, and the shorter peak obtained at a lower wavelength corresponding to the axial surface plasmon resonance. The dominant longitudinal peak can be tuned by carefully controlling the GNR synthesis procedure [34]. GNR formulations, corresponding to longitudinal peak of 840 nm, were synthesized using the seed-mediated procedure. The plasmon peak as designed to overlap with the blue edge of the OCT imaging spectral band is shown in Figure 1, to provide a steep wavelength-dependent response [35]. TEM imaging of the GNRs corresponding to a longitudinal peak of 840 nm shown in Figure 1 (inset), reveals rod-shaped well-dispersed nanoparticles 45 ± 6 nm in length and 12 ± 2 nm in width. 

### 3.2. Gold Nanorod Surface Modification and Antibody Functionalization

Active groups were generated on the surface of GNRs to act as anchors for the covalent attachment of target specific antibodies. Zeta potential measurements were conducted on the GNRs dispersed in DI water before and after each step of the bio-conjugation procedure. The change in zeta potential measurements, as shown in Table 1, also served as an indirect method to confirm the success of each conjugation step. Initially, the CTAB coated GNRs displayed a positive zeta potential of +38.8 ± 2, which is attributed to the presence of cationic quaternary ammonium groups [36]. On modification of GNRs with PAA the zeta potential measured was −64.9 ± 5 which can be attributed to the presence of anionic COO^−^ groups on the GNR surface. Anti-Glut-1 antibodies were then linked to the modified GNRs using carbodiimide chemistry. Following the antibody-modified GNR linking procedure the excess or unbound antibodies separated using ultra-centrifugation were analyzed using optical spectroscopy at a wavelength of 280 nm. The peak height of the optical spectroscopy signal obtained is directly proportional to the concentration of anti-Glut-1 in the supernatant. Based on the optical spectroscopy measurements conducted at 280 nm and 840 nm respectively we were able to determine that 23.45 μg/mL of anti-Glut antibodies bound to 193.45 × 10^14^ GNRs. Based on these results the surface density (number of antibodies bound per GNR) was calculated to be 13.25. In order to determine the bio-activity of these bio-conjugated antibodies a modified ELISA along with horseradish peroxidase labeled anti-rabbit secondary antibody was utilized. Table 2, shows the average absorbance measurement recorded for three distinct 96 well-plates. The secondary antibody binds specifically to the GNR tagged primary anti-Glut-1 antibodies. The statistically different results allow us to conclude that the GNR tagged antibodies bind selectively to Glut-1, thus maintaining their bioactivities. 

**Table 1:** Zeta potential measurements of gold nanorods recorded before and after surface modifications.

**Surface activity of Nanorods **	**Zeta potential (ζ) mV**
(suspended in DI water)	
CTAB	+38.8 ± 2
Anionic polymer (Poly-acrylic acid)	−64.9 ± 5
Anti-Glut-1 labeled GNRs	−47.36 ± 3

**Table 2:** Average optical absorbance measured using plate reader with a 750 nm emission filter for each well.

	Empty	Glut-1	Bare	Glut-1	Glut-1	Glut-1	Glut-1	Glut-1	Glut-1	BSA	BSA	BSA
	well	Antibody	GNR	protein	protein	protein	protein	protein	protein			
					Ab	Bare GNR	Ab-GNR	Ab	Ab-GNR	Ab-GNR	Bare GNR	
								2'-Ab	2'-Ab			2'-Ab
Average Absorbance	0.595	0.555	0.544	0.619	0.562	0.560	0.544	0.885	0.719	0.552	0.535	0.600

### 3.3. Glut-1 Detection Using OCT

**Figure 2:**
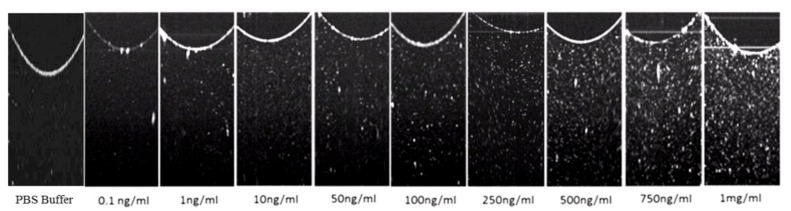
Background subtracted OCT images of well plates containing anti-Glut-1 tagged GNRs attached to Glut-1 protein suspended in PBS.

**Figure 3:**
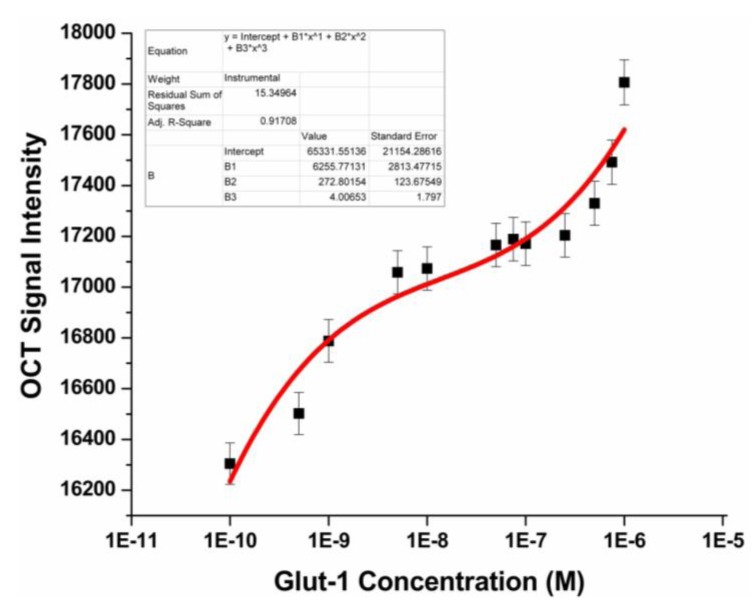
Calibration curve depicting averaged OCT signal intensity *versus* corresponding initial concentration of Glut-1 protein.

After optimizing the key steps in the molecular architecture of the sensor, we assessed the performance of the immunosensor by challenging it with increasing concentrations of Glut-1 protein from 0.1 ng/mL to 1 mg/mL. The OCT images of the sonicated 96-well plates containing an initial Glut-1 concentration of 0.1 ng/mL, 1 ng/mL, 10 ng/mL, 50 ng/mL, 100 ng/mL, 250 ng/mL, 500 ng/mL, 750 ng/mL, and 1 mg/mL are shown in Figure 2, PBS buffer acts as the negative control. A thorough evaluation of Figure 2, can allow us to discern that the scattering visible in the OCT image due to the presence of GNRs intensifies in proportion to the concentration of Glut-1. The OCT images were then normalized and, by choosing a region of interest, the average OCT signal intensity was calculated for each image. A calibration curve was plotted using the average OCT signal intensity (four replicates × three distinct plates = 12 data points/Glut-1 concentration) as shown in Figure 3. Based on the calibration curve we can conclude that the nano-optical protein measurement sensing strategy developed is capable of detection Glut-1, within a detection range of 10 ng/mL to 1µg/mL of Glut-1. The sensitivity of this assay, or lower limit of detection was defined as the lowest protein concentration that could be differentiated from the negative control (PBS buffer). The sensitivity of the assay was measured to be 10 ng/mL. The nano optical sensing strategy described provides a wide detection range as compared to the commercially available Glut-1 ELISA kits which perform within a range of 0.312 ng/mL–20 ng/mL (USCN Life Science Inc.), 1.15 ng/mL–20 ng/mL (Labome) and 1.15 ng/mL–20 ng/mL (Cusabio Biotech Co., Ltd.). Spherical gold nanoparticles have also been used for the development of similar biosensors based on the principles of exploiting the plasmonic properties of gold. Maier *et al*., describe the development of an optical immunochip based sensor for the detection allergens in food matrices using spherical gold nanoparticles. The single step immunoassay displays a 10 ng/ml detection limit for the measurement of ovalbumin [19]. Thanh *et al*. developed an aggregation based immunoassay for anti-Protein A using spherical gold nanoparticles which displays a dynamic range of two orders of magnitude and a limit of detection of 1 μg/mL [16]. 

### 3.4. Specificity Study

**Figure 4:**
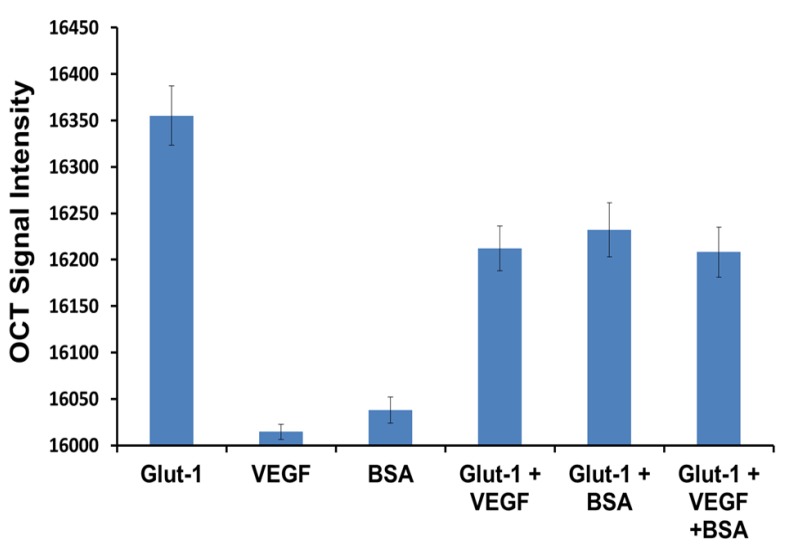
Specificity study conducted using human vascular endothelial growth factor (VEGF) and BSA as competitive analytes for anti-Glut-1 tagged gold nanorods.

Specificity is a crucial parameter, which influences the performance of a biosensor in real matrices. We need to prove that the presented sensor responds only to the Glut-1 and anti-Glut-1 immunoreaction and not towards the nonspecific interaction with other proteins. In order to demonstrate the specificity of the biosensor we conducted studies using VEGF and BSA as competitive analytes. Figure 4, shows the average OCT signal intensity (10 replicates were conducted for each data set) measured for Glut-1, VEGF and BSA respectively. The wells containing Glut-1 display the highest OCT signal intensity, which translates to the highest binding efficiency of anti-Glut-1 tagged GNRs. The wells containing VEGF and BSA show a non-significant increment of 12.65 ± 8.3 and 36.35 ± 14.1 respectively in signal intensity as compared to control wells containing PBS buffer. Hence we can conclude that the 353.13 ± 32.1 signal intensity increment measured for the wells containing Glut-1 was caused due the highly selective immunoreactions between Glut-1 and anti-Glut-1 tagged GNRs. In order to study the interference that could be caused by the interference of non-specific proteins in the test sample we tested wells containing combinations of Glut-1 + VEGF, Glut-1 +BSA and Glut-1 + VEGF +BSA such that the combined molarity of the samples was 500 ng/mL. Based on the results shown in Figure 4 we can conclude that the presence of VEGF and BSA did not cause any hindrance towards the binding of GNRs to Glut-1 protein. 

## 4. Conclusions

In this work we have successfully designed and characterized a nanoplasmonic immunosensing system for the rapid detection of protein biomarkers such as Glut-1. The immunosensor displays a wider detection range of 10 ng/mL to 1 µg/mL for Glut-1, as compared to commercially available ELISA kits. The sensing system requires only one incubation step which results in fewer washes and shorter analysis time as compared to traditionally used assays. The use of antibody conjugated GNRs as molecular labels allows measurements requiring no substrate development and stability over long time periods due to non-photobleaching and non-degradation of label. A few disadvantages of the technique are that the detection limit in the case of our model analyte Glut-1 is higher than the traditional ELISA. The working principle also requires that the GNRs be suspended in solution, which warrants the use of sonication to break up the attachment of model analyte protein Glut-1 from the bottom of the well plates. The immune-sensing strategy described using Glut-1 as a model analyte can be applied towards the measurement of other protein biomarkers of interest by selecting the appropriate recognition molecule such as antibody, Fab fragment or aptamer. Our future work involves the development of a microfluidic platform based on a similar principle of detection, which would enable better detection limit, shorter sonication time, and point-of-care measurement capabilities. 
